# *Prosopis alba* Seed as a Functional Food Waste for Food Formulation Enrichment

**DOI:** 10.3390/foods11182857

**Published:** 2022-09-15

**Authors:** Florencia M. Correa Uriburu, Florencia Cattaneo, Luis M. Maldonado, Iris C. Zampini, María R. Alberto, María I. Isla

**Affiliations:** 1 Instituto de Bioprospección y Fisiología Vegetal (INBIOFIV-CONICET-UNT), San Lorenzo 1469, San Miguel de Tucumán 4000, Tucumán, Argentina; 2 Instituto Nacional de Tecnología Agropecuaria, Estación Experimental Agropecuaria, Famaillá, PROAPI, Ruta Provincial 301-km 32, Famaillá 4132, Tucumán, Argentina; 3 Biolates Network for Sustainable Use of Ibero-American Vegetable Biomass Resources in Cosmetics (BIOLATES, CYTED), San Miguel de Tucumán 4000, Tucumán, Argentina; 4 Facultad de Ciencias Naturales e Instituto Miguel Lillo, Universidad Nacional de Tucumán, San Lorenzo 1469, San Miguel de Tucumán 4000, Tucumán, Argentina; 5 Instituto de Biotecnología Farmacéutica y Alimentaria (INBIOFAL-CONICET-UNT), Av. Kirchner 1900, San Miguel de Tucumán 4000, Tucumán, Argentina

**Keywords:** phenolic enriched extracts, antioxidant activity, macronutrients, phytochemicals, bioactive compounds

## Abstract

The present study describes how flour and phenolic enriched extracts (PEE) are obtained from seed (food waste) of 10 different *P. alba* (algarrobo blanco) clones and their characterization to be used as non-conventional sources of potential functional ingredients. Seed flour and PEE obtained from Argentinian *P. alba* cultivars were chemically characterized. The antioxidant capacity was also determined. The results showed variability in macronutrient composition of seed flour obtained from different clones. Among them, seed flour obtained from P4, P5, P6, P10, P12, and P13 clones showed a higher protein and fiber content than the other clones. On the other hand, PEE obtained from P6, P7, and P10 clones showed the highest content of phenolic component (7.32–8.58 mg GAE/g flour). The extracts obtained from them also showed high antioxidant activity (scavenging activity on ABTS^•+^, HO^•^, and H_2_O_2_). *C*-glycosyl flavones, including vicenin II, isoschaftoside, schaftoside, vitexin, and isovitexin were the major components extracted in all clones. These compounds have remarkable properties for disease prevention linked to oxidative stress. Therefore, the *P. alba* seed could be considered as functional food waste with a great potential to be used as a novel renewable and sustainable material for the production of bioactive food formulations.

## 1. Introduction

*Prosopis* includes 45 species that belong to the Mimosoideae subfamily within the Fabaceae family, distributed in America, Asia, and Africa [1]. *Prosopis* grows easily in tropical, subtropical, and semi-arid soils, enduring high temperatures, drought, alkalinity, and soil salinity [2]. In Argentina, there are 27 biotypes of *Prosopis* spp. [3]. In our country, 100,000 tons of *Prosopis* wood is harvested annually, to be used in the making of doors, floors, blinds, and windows [4,5]. On the other hand, *Prosopis* pods have been used since pre-Hispanic times to feed both livestock and human. They are used to prepare drinks (añapa, aloja, and chicha), and numerous foods such as syrups, flour, and sweets (jam, arrope, patay) [6]. In fact, *P. alba* whole pod flour (Article 681 tris) and *P. alba* seed flour (Article 681) are included in the Argentine Food Code [7]. Until now, *Prosopis* flours were obtained from whole pods, although in this process the seed were discarded as waste. The pods flours were evaluated functionally and chemically and its properties, i.e., antioxidant, hypoglycemic, antilipase, and anti-inflammatory activities were demonstrated, but the *P. alba* seed flours were not yet fully characterized [1,5,8,9,10,11,12,13,14]. The production of *P. alba* whole pod flour is growing, thus increasing the generation of biomass that should be used. In this sense, an important aspect regarding economic benefit is to recover the high-added-valued constituents present in fruit processing wastes. The availability of this vegetable raw material, together with the problem represented by the accumulation of waste and its unexploited potential, has encouraged researchers to develop projects on the value-addition potential of fruit processing waste, such as enrichment in food formulation.

One of the main difficulties faced by the industrialization of *P. alba* flour is its high variability, for both biomass and pod production [15]. Regarding progenies, this happens because *Prosopis* are pollinated by insects and are self-incompatible. Therefore the seeds of the same tree are highly likely to have multiple crosses. In this way, clonal propagation is an essential tool for the genetic improvement of *P. alba*. Fortunately, it can be propagated clonally by grafting, rooting the cuttings, and air layering [15]. Clonal selections of *P. alba* have been made for its economically valuable characteristics, such as rapid growth rate, high pod production, and improved flavor (sweeter pods without bitter characteristics) [15,16]. Progeny trials of elite trees have been conducted in Santiago del Estero (27°45′ S, 64°15′ W) with seeds of 57 individual trees from 8 regions of Argentina. The initial selection was based on productivity (biomass) and pod taste (sweet or very sweet, astringent or very astringent). The plants that produced sweet or very sweet pods were cloned by grafting stems from mature trees. The rejuvenated clones (10) were multiplied by rooting cuttings [15,16,17].

The present study describes for the first time the chemical characterization and antioxidant capacity of flour and phenolic enriched extracts (PEE) obtained from seeds of previously selected *P. alba* clones.

## 2. Materials and Methods

### 2.1. Plant Material

**Figure 1:**
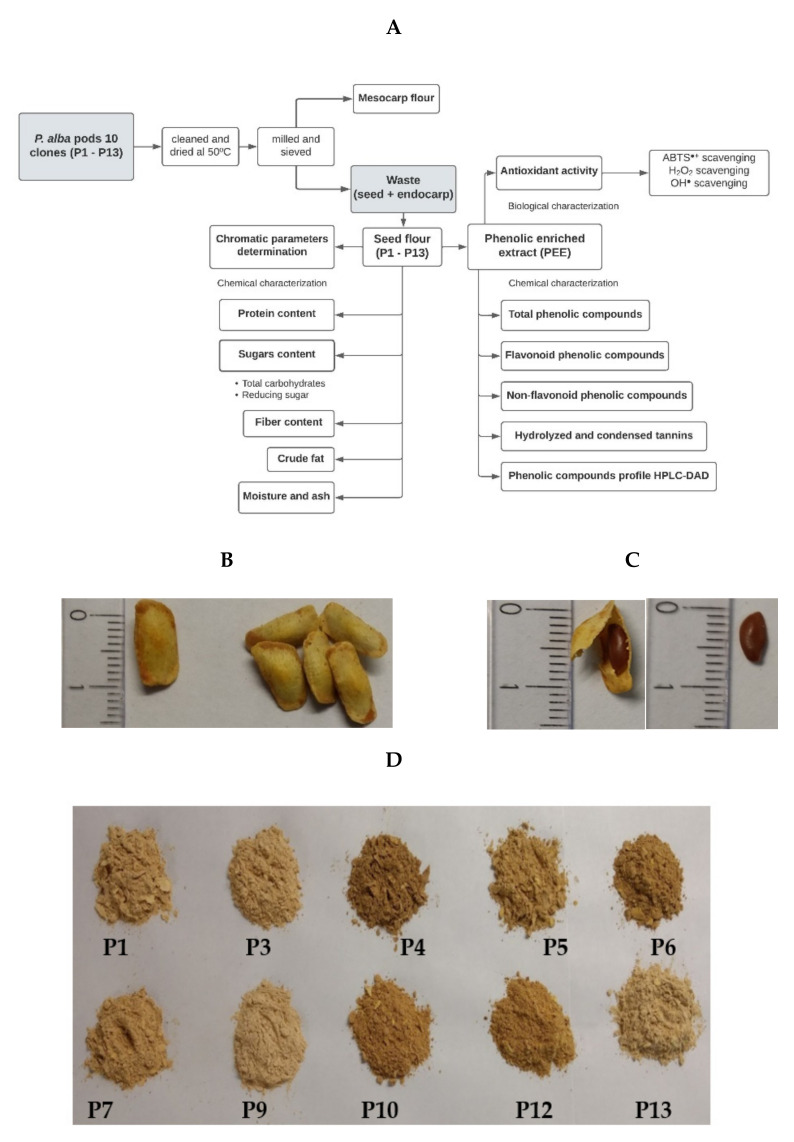
(**A**) Flowchart of the methodology. (**B**) Seeds with endocarp. (**C**) Seeds and (**D**) flour of seeds from 10 clones of *Prosopis alba* (P1–P13).

The mature pods of 10 clones of *Prosopis alba* were collected in an experimental field of the Universidad Católica de Santiago del Estero in a town called Fernández, in the province of Santiago del Estero, Argentina. Figure 1A shows a summary of the methodology.

### 2.2. P. alba Seed Flour Preparation and Chromatic Parameter Determination

The pods with the same ripening stage were cleaned so as to remove any strange material and dried in a forced-air oven at 50 °C until reaching constant weight. The pods were named P1 to P13. The pods P2, P8, and P11 were discarded because they had abundant polluting plant material. The dried pods were milled and then sieved to separate both the mesocarp flour and whole seeds with endocarp (considered as waste). The seeds (Figure 1B,C) were processed with a knife mill to obtain the fine flour (≤149 μm) of each sample (seed flours P1–P13), as shown in Figure 1D. The seed flour was stored under vacuum at −20 °C until use. The chromatic parameters of the flour were measured with a Chroma meter CR-400 (Konica Minolta, Tokyo, Japan) colorimeter using the CIELab system, the results being expressed as chromaticity coordinates L*, a*, and b* (objective parameter). The coordinated L* represents lightness (contribution of black or white varying between 0 and 100); a* represents the contribution of green or red (negative or positive), and b* represents the contribution of yellow or blue (negative or positive) [18].

### 2.3. Determination of Chemical Composition

#### 2.3.1. Macronutrient Determination

A differential extraction from *P. alba* seed flour was carried out to obtain proteins and sugars [19]. Crude protein content was determined by using the Kjeldahl method [20]. Total carbohydrates and reducing sugar were measured using the method of Orqueda et al., 2017 [21]. Crude fat was determined with a Soxhlet apparatus, extracted with petroleum ether (40–60 °C) for 4 h according to AOCS, 1989 [22]. Results were expressed as mg of macronutrient/g of flour. The moisture content was determined by gravimetric method between the fresh and the dry samples [23]. The latter were incinerated in a muffle (500 °C) and, then, the ashes were weighed on an analytical scale to determine the ash content [24].

#### 2.3.2. Fiber Analysis

Fiber content was determined by using acid and alkaline digestion and heating [19]. Crude fibers were calculated as the difference in weight before and after incineration in muffle. Results were expressed as g of fiber/100 g of flour.

#### 2.3.3. Phenolic Enriched Extracts: Characterization

Five g of *P. alba* seed flour was mixed with 50 mL of 70° ethanol in an ultrasonic bath for 10 min at 25 °C. Then, the samples were placed in a shaker for 24 h at 25 °C and 120 rpm. Then, each sample was vacuum filtered to obtain the phenolic enriched extract (PEE). Then, the PEE was dried under reduced pressure at 40 °C and freeze-dried. The powder obtained was stored at −20 °C until use. Total phenolic compounds content was determined by using Folin-Ciocalteu reagent (Merck, Darmstadt, HE, Germany) [25] and was expressed as mg of gallic acid equivalent (GAE)/g flour.

Total non-flavonoid phenolic compounds were determined by using the method described by Isla et al. (2014) [26]. First, flavonoids were precipitated with formaldehyde (8 g/L). Then, the samples were centrifuged at 9000× *g* for 5 min (Rolco, Buenos Aires, Argentina), and non-flavonoid phenolic compounds were quantified in the supernatant with the Folin–Ciocalteau reagent. Flavonoid phenolic compounds were calculated by using the difference between the total phenolic and non-flavonoid phenolic content. Flavone and flavonol content were determined by using the aluminum chloride colorimetric method [21]. Hydrolyzed and condensed tannins were extracted and quantified as described by Costamagna et al. (2013) [19].

#### 2.3.4. Profile of Phenolic Compounds Using HPLC-DAD

The chromatographic profiles of PEE were obtained by HPLC coupled to a diode array detector (HPLC-DAD) (Waters Corporation, Milford, MA, USA) in an analytical C18 column (XBridge) by using a linear gradient solvent system consisting of 0.1% acetic acid in water (A) and 0.1% acetic acid in methanol as follows: 90% to 43% A for 45 min, 43% to 0% A for 45 to 60 min, holding 100% B for 5 min. The injected sample volume was 20 µL with a flow rate of 0.5 mL/min. The identification of phenolic compounds was carried out by comparing the retention times and spectral data (220–600 nm) of each peak with those of standards (HPLC quality) from Sigma-Aldrich (St. Louis, MO, USA) and Fluka Chemical Corp. (Ronkonkoma, NY, USA).

### 2.4. Antioxidant Activity

#### 2.4.1. ABTS^●+^ Scavenging Activity

ABTS^●+^ scavenging activity was measured [27] on a microplate reader by using 200 µL of ABTS^●+^ solution and 100 µL of sample to final concentrations between 2 and 10 µg GAE/mL of phenolic compounds. Absorbance was measured at 1 and 6 min at 734 nm. The sample concentration required to scavenge 50% ABTS^●+^ was determined as 50% ABTS^●+^ scavenging capacity (SC_50_). Results are expressed in µg gallic acid equivalent (GAE)/mL of PEE.

#### 2.4.2. Hydrogen Peroxide Scavenging Activity

Hydrogen peroxide (H_2_O_2_) scavenging activity of PEE obtained from seed flours was carried out through the optimized enzymatic colorimetric assay [28] adapted to a microplate assay. Briefly, different sample concentrations diluted in 84 mM sodium phosphate buffer (pH7) reacted with H_2_O_2_, phenol, and 4-aminoantipyrine reaction system in the presence of horseradish peroxidase (HRP), at 37 °C. This reaction was measured at 504 nm. SC_25_ values denote the sample concentration required to scavenge 25% H_2_O_2_. Results are expressed in µg gallic acid equivalent (GAE)/mL.

#### 2.4.3. Hydroxyl Radical Scavenging Activity

The hydroxyl radical scavenging activity of PEE was determined according to Cattaneo et al. (2016) [10]. The reaction mixture contained 2-deoxy-D-ribose/FeCl_3_ and different extract concentrations (up to 20 µg GAE/mL) with and without EDTA. The reaction was started with H_2_O_2_ and ascorbic acid at 37 °C for 60 min. Then, 2-thiobarbituric acid was added and incubated for 20 min at 100 °C. Absorbance was recorded at 532 nm. Results are presented as SC_50_ values in µg GAE/mL required to inhibit the degradation of 50% 2-deoxy-D-ribose. Quercetin (5–50 µg/mL) was used as a reference compound.

### 2.5. Statistical and Chemometric Analysis

The comparison of mean values was made by ANOVA, followed by a Tukey post-test. A value of *p* ≤ 0.05 was considered significant.

The chemical components concentration and the SC_50_ values of free radicals by several methodologies for each flour sample were exported in a matrix of 14 columns and 30 rows, where each column represents a variable and each row represents a clone. Principal component analysis (PCA) and hierarchical clusters (HCA) using the Infostat software version 2015 [29] (Universidad Nacional de Córdoba, Córdoba, Argentina) were applied. PCA scaling and centering were used. Mean values were used for graphs, where each point on the graph corresponded to the mean value for each clone. HCA was performed by calculating the Euclidean distance and considering average link clustering as a method for calculating the distance between clusters. In addition, data was standardized. The number of groups was determined considering the cut-off line half the Euclidean distance.

## 3. Results and Discussion

Clones of *P. alba* were identified in a progeny trial established in 1990 in Santiago del Estero (Argentina) [15]. The trees showed rapid growth, high pod production, and sweet pods. All clones had good sensory properties for use in human food [15,16]. The pods of 10 clones were milled to obtain mesocarp flour. The discarded seeds in the pod milling process were submitted to grinding to obtain seed flour. Seed flour chromatic values were determined to compare all clones. The values were L* = 58.41 ± 10 to 60.40 ± 10; a* = 3.42 ± 1 to 8.38 ± 1 and b* = 33.43 ± 2 to 35 ± 2. Similar results were obtained to *P. alba* mesocarp flour [11]. The flours P4, P5, P6, P10, and P12 showed the highest values of a* (8.38; 7.20; 7.32; 7.30; 7.10, respectively), as shown in Figure 1D. This parameter showed the highest chromatic variability between clones.

### 3.1. Macronutrients

The carbohydrate content of seed flour exhibits the total carbohydrate content between 5.16 g/100 g and 23.15 g/100 g. P6, P7, P9, and P10 showed the highest sugar content. The total sugar content of seed flour was lower than that of mesocarp flour (73.95 g/100 g), data reported by Cardozo et al., 2010 [1] (Table 1). The mesocarp of pods belonging to *Prosopis* species is rich in sugars [5,13,30] and the seed is rich in protein [9,10,30]. Besides, P10 showed the highest content of reducing sugar.

The protein content (Table 1) of *P. alba* seed flour varied between 7.98 and 13.34 g/100 g. P4 and P5 clone seed flours showed even greater total protein content than *P. alba* mesocarp flour [1]. Protein values were similar to amaranth seed, with values between 13.2 and 18.2% [31]. According to guidelines for the use of nutrition and health claims of the Codex Alimentarius of FAO (Food and Agricultural Organization, 2011) [32], when a solid sample has a protein content ≥10% it could be considered a source of protein. All *P. alba* seed flours showed this condition; only P3 and P9 clones exhibited less than 10% protein values. Fat values were lower in seed flour (1.70 to 3.98 g/100 g) than in mesocarp flour (11.3 g/100 g) [1]. P4–P6 clones showed the highest fat content (Table 1).

Crude dietary fiber content varied between 13.90 and 27.69 g/100 g. P1, P4, and P5 showed the highest crude fiber content. The deficiency of dietary fiber in the human diet has been associated with constipation, diverticulosis, cardiovascular diseases, and cancer, so its study is important [33]. In addition, some *P. alba* seed flours had a higher fiber content than that reported for mesocarp and cotyledon flour of *P. alba* [1,10]. Ash determines the nutritional quality of food, affecting the rheological properties and cooking quality [34]. P4 flour showed the highest ash content (4.25%).

The low moisture content (less than 10%) would improve its storage stability by preventing mold growth and other biochemical reactions and extending the final product shelf life. All seed flours have this condition, except for P10 and P12 (11.77% and 12.50%, respectively).

### 3.2. Secondary Metabolites

The secondary metabolites play an important role in the defense systems of plants against environmental stress and pathogenic attacks. In addition, these compounds have various biological activities, so they are used as medicinal, food, and cosmetic ingredients [35]. There are some studies regarding polyphenols in *P. alba* cotyledons, seeds, and mesocarp flour [8,10,12,13,14]. Table 2 shows the phytochemical composition of 10 clones of *P. alba*. The total phenolic compound content extracted from *P. alba* seed flour was between 5.05 and 8.58 mg GAE/g of flour. These values are higher than that obtained by Sciammaro et al. (2016) [13] (3 mg GAE/g DW) for *P. alba* seeds collected in Ingeniero Juárez, Formosa in the northeast of Argentina, but were lower than the total phenolic content of *P. alba* cotyledon flour (11 mg GAE/g of flour) reported by Cattaneo et al., (2016) [10] for *P. alba* collected in Amaicha del Valle (Tucumán, Argentina). Flours of P6, P7, and P10 clones showed the highest phenolic content, higher than *P. alba* mesocarp flour (4.6 mg GAE/g flour), [8,14] and *Ceratonia siliqua* pulp flour [36]. Non-flavonoid phenolic compounds were the main phenolic components of *P. alba* seed flour (2.68 to 4.01 mg GAE/g of flour). P10 flour showed the highest flavones and flavonol content. The flavonoid content was between 0.098 and 0.308 mg of quercetin equivalent (QE)/g flour (mg) and was lower than those found in *P. alba* cotyledons flour (3.9 mg EQ/g flour) and mesocarp flour (33–87.5 mg EQ/g flour) [1,8,10,14].

Regarding the content of condensed tannins, the values varied between 44.75 and 223.73 mg of procyanidin B2 equivalent (PB_2_E)/100 g flour. The highest content was observed in P10, P12, and P13 clones. P7 presented a similar content of these compounds to that reported to *P. alba* cotyledon flours (175 mg PB_2_E/100 g flour) [10].

### 3.3. HPLC Analysis

**Figure 2:**
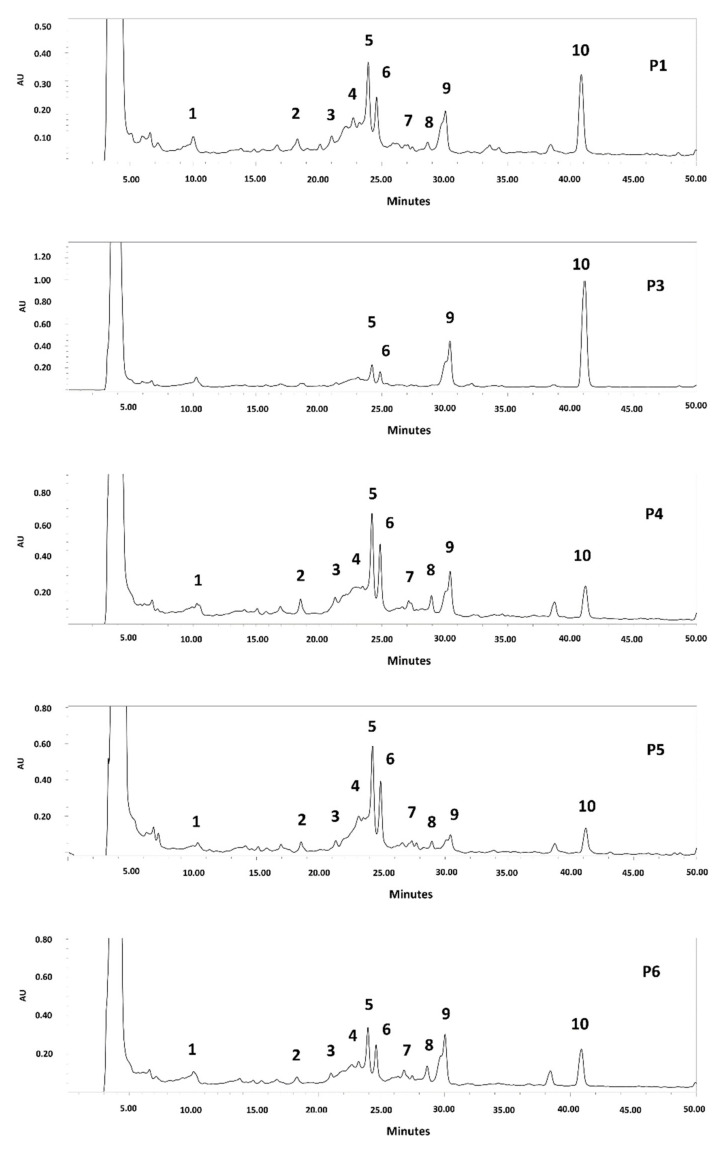
HPLC chromatogram of polyphenols from *P. alba* seed flour (Clones P1–P6). Detection: UV_254_ nm. Compounds: 1: isoschaftoside hexoside; 2: schaftoside hexoside; 3: Vicenin II (apigenin-di-*C*-hexoside)/isomer; 4: vicenin II/isomer; 5: isoschaftoside (apigenin-*C*-hexoside-*C*-pentoside); 6: schaftoside; 7: unknown; 8: vitexin; 9: isovitexin; 10: unknown.

Since an extract rich in polyphenols was obtained from the seeds of all the clones, it was decided to determine their phenolic compound profile. The HPLC chromatogram is shown in Figure 2 and Figure S1 (Supplementary Materials). *C*-glycosyl flavones, including vicenin II, isoschaftoside, schaftoside, vitexin, and isovitexin, were identified in the seed extracts of all analyzed clones. The same results were reported for *P. alba* and *P. nigra* mesocarp flour [8,11,12,13,14,37,38] and *P. nigra* seed [39]. Other authors have reported several biological activities for apigenin-C glycosides, such as antioxidant, anti-inflammatory, anti-cancer, anti-platelet activity, angiotensin converting enzyme inhibition, protective effects against neurological and psychiatric diseases, protective activity in the cardiovascular system, protective effects against diseases of the endocrine system and metabolic, anti-microbial, and antiviral effects, among others [38,39,40]. In addition, they are rapidly absorbed after oral administration and are distributed by plasma in different tissues [40].

### 3.4. Biological Activities

This study analyzed the antioxidant activity of PEE obtained from seed flour (Table 3). The samples exhibited ABTS^•+^ reducing capacity with SC_50_ values between 4 and 6.7 µg GAE/mL. The antioxidant capacity of PEE obtained from seed flour was higher than *P. alba* mesocarp flour PEE (13 µg GAE/mL) [11]. The ABTS^•+^ scavenging activity by the PEE was lower than that of a commercial natural antioxidant such as quercetin (SC_50_ = 1.4 µg/mL) but similar to BHT, a synthetic antioxidant used in the food industry (SC_50_ = 3.52 µg/mL).

The PEE of *P. alba* seed flour also showed scavenging activity of H_2_O_2_ with SC_25_ values between 5.9 and 13.6 for the most active extracts and between 20.5 and 23.3 µg GAE/mL for the less active extracts. Scavenging activity of HO^•^ was also found with SC_50_ values lower than those found for PEE from *P. alba* mesocarp flour [10]. Phenolic extract antioxidant activity could be attributed to *C*-glycosyl flavonoids, compounds with demonstrated antioxidant capacity [8,11,37,38,39,40].

### 3.5. Principal Component Analysis

**Figure 3:**
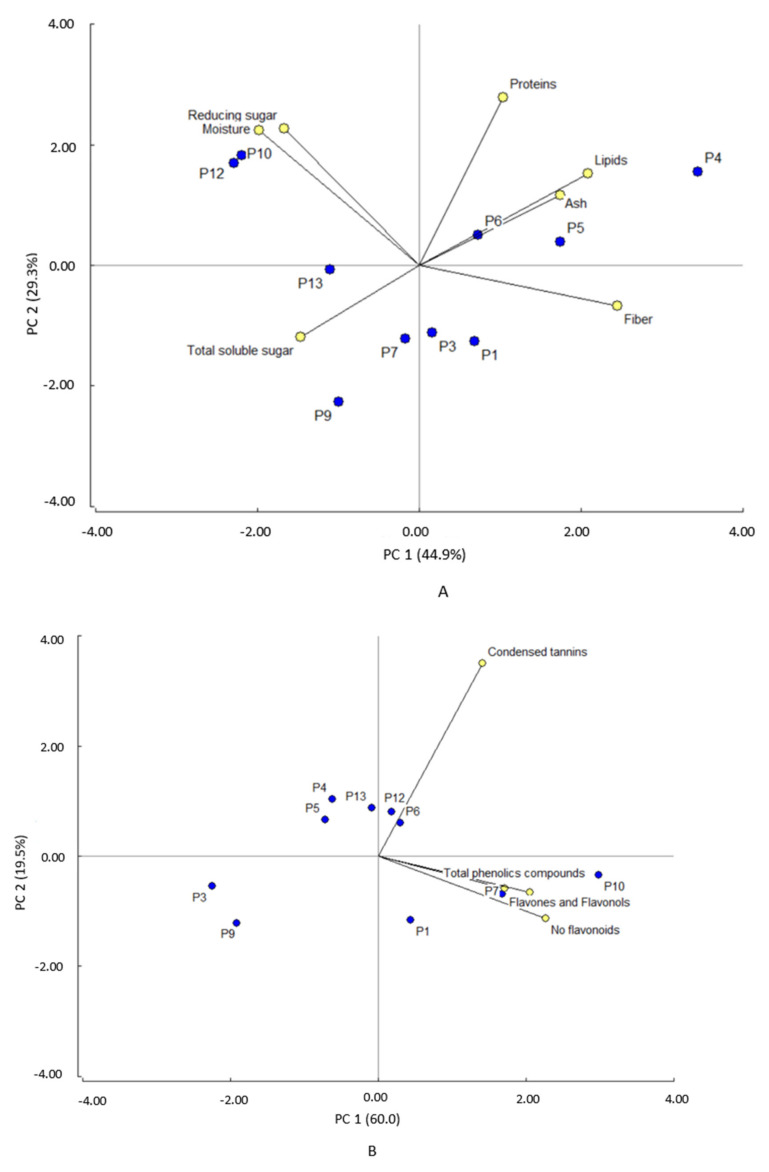
Principal component analysis for (**A**) proximal components and (**B**) phytochemicals.

Considering the macronutrient components as variables, the principal component analysis (PCA) showed two components that explain 74.2% of the variability (Figure 3A). Principal component 1 (PC1) explains 44.9% of the variability and is positively influenced by fibers and lipids and negatively by moisture. This component clearly separates P4, P5, P6, and P1 clones (+) from P12, P10, P13, and P9 clones (−). The first group showed high fiber and lipid content and low moisture content (Table 1), whereas the second group presented low lipid and fiber level and high moisture content. Principal component 2 (PC2) explains 29.3% of the variability and is influenced by protein and reducing sugars. This component clearly separates P12, P10, and P4 clones from P9, P7, P3, and P1 clones. The former group has higher protein content, reducing sugars and moisture than the latter group. Considering phytochemicals composition as a variable for the PCA analysis, the principal component analysis (Figure 3B) showed that the two components explain 79% of the variability. PC1 explains 60% and is influenced by the content of both non-flavonoid phenolic compounds and total phenolic compounds. This component separates P10 and P7 clones (+) from P9 and P3 clones (−). PC2 explains 19.5% of the variability and is influenced by condensed tannin content. This component separates P1, P9, P3, P7, and P10 clones from P4, P5, P6, P12, and P13 clones. The latter group showed the highest content of condensed tannins.

### 3.6. Hierarchical Cluster Analysis

**Figure 4:**
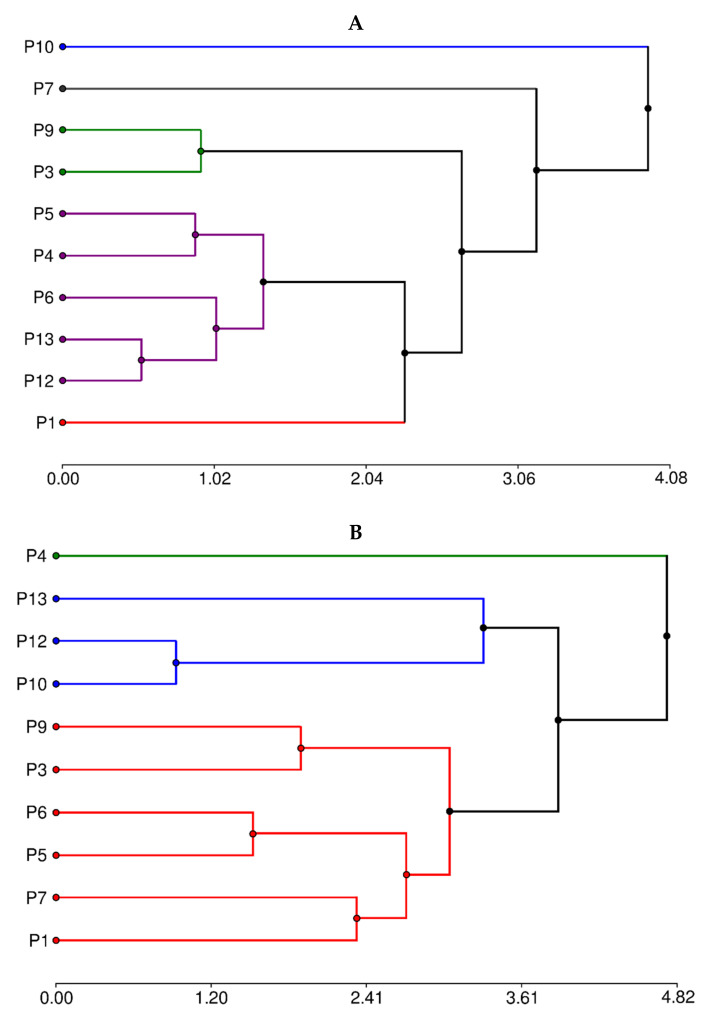
Hierarchical clusters analysis by (**A**) secondary metabolites composition and (**B**) proximal composition.

When attempting to determine the groups of secondary metabolites, two well-marked—besides three individual ones—appear (Figure 4A).

The group with the highest number of individuals (P4, P5, P6, P12, and P13) is determined by the highest condensed tannin concentration. The second group, P9 and P3, is characterized by the lowest content of all phytochemicals. On the other hand, clones P1, P7, and P10 are each considered to correspond to a different group. P10 showed the highest content in flavones and flavonols, total phenolic, and non-flavonoids. P1 and P7 showed lower values of these compounds concerning P10 but higher than the other groups.

Hierarchical cluster analysis by proximal composition (Figure 4B) suggests three groups. The group formed by P10, P12, and P13 is determined by the low concentration of fiber and lipids. The P4 clone showed the highest lipid, protein, and ash concentration. The third covered the rest of the clones and represented the samples with intermediate values of all components.

## 4. Conclusions

The results obtained in this study established that principal component analysis and hierarchical cluster analysis were efficient tools for clone selection according to the chemical composition and functional properties of *P. alba* fruit waste.

Seed flour obtained from P4, P5, P6, P10, P12, and P13 clones showed a higher protein and fiber content than the other clones. On the other hand, PEE obtained from P6, P7, and P10 showed the highest content of phenolic component, mainly *C*-glycosyl flavones and high antioxidant activity.

Considering the potential use of seed flour for nutritional properties or phytochemical extraction, the selection of adequate clones is possible. Therefore, both the flour and the PEE obtained from *P. alba* fruit wastes have a great potential to be used as a novel renewable and sustainable material for the production of bioactive food formulations.

## Figures and Tables

**Table 1:** Proximal composition of the seed flour from 10 clones of *Prosopis alba*.

Macronutrient Content (g/100 g Flour)	P1	P3	P4	P5	P6	P7	P9	P10	P12	P13
Total carbohydrates	11.06 ± 2.04 ^c,d,e^	14.47 ± 2.26 ^b,c,d^	5.16 ± 0.58 ^e^	12.35 ± 1.75 ^c,d^	17.29 ± 3.04 ^a,b,c^	23.15 ± 1.77 ^a^	21.10 ± 5.54 ^a,b^	17.07 ± 0.28 ^a,b,c^	16.95 ± 0.11 ^a,b,c^	7.83 ± 0.34 ^d,e^
Reducing sugars	1.14 ± 0.27 ^e^	2.53 ± 0.13 ^c,d^	1.89 ± 0.39 ^d,e^	1.97 ± 0.22 ^d^	3.21 ± 0.08 ^c^	1.89 ± 0.18 ^d,e^	1.94 ± 0.19 ^d,e^	5.78 ± 0.46 ^a^	4.77 ± 0.37 ^b^	1.83 ± 0.33 ^d,e^
Total proteins	10.76 ± 0.04 ^d^	9.61 ± 0.08 ^e^	13.07 ± 0.08 ^a^	13.34 ± 0.26 ^a^	12.27 ± 0.01 ^b^	10.82 ± 0.03 ^d^	7.98 ± 0.03 ^f^	11.79 ± 0.47 ^b,c^	12.33 ± 0.56 ^b^	11.08 ± 0.10 ^c,d^
Fat	2.82 ± 0.11 ^b^	1.97 ± 0.38 ^b^	3.98 ± 0.09 ^a^	3.08 ± 0.09 ^a,b^	3.25 ± 0.07 ^a,b^	1.74 ± 0.65 ^a,b^	1.70 ± 0.67 ^b^	2.63 ± 0.34 ^b^	2.28 ± 0.20 ^b^	1.79 ± 0.07 ^b^
Fiber	22.45 ± 0.06 ^b^	17.73 ± 0.35 ^c^	27.69 ± 1.05 ^a^	25.85 ± 1.92 ^a^	21.35 ± 0.61 ^b^	17.39 ± 0.34 ^c^	15.75 ± 0.24 ^c^	15.46 ± 0.21 ^d^	13.90 ± 1.05 ^d^	16.12 ± 0.48 ^e^
Ash	2.99 ± 0.04 ^d^	3.80 ± 0.04 ^b^	4.25 ± 0.07 ^a^	3.69 ± 0.02 ^b^	3.70 ± 0.01 ^b^	3.12 ± 0.01 ^d^	3.47 ± 0.02 ^c^	3.37 ± 0.16 ^c^	3.32 ± 0.06 ^c^	3.32 ± 0.05 ^c^
Moisture	6.19 ± 0.67 ^g,h^	6.17 ± 0.04 ^h^	6.92 ± 0.46 ^e,f^	6.51 ± 0.02 ^g,h^	7.42 ± 0.32 ^d^	6.56 ± 0.42 ^b,f^	7.10 ± 0.18 ^d,e^	11.77 ± 0.08 ^b^	12.50 ± 0.26 ^a^	9.16 ± 0.31 ^c^
g seed flour/100 g pod	62.76 ± 6.28 ^b^	70.76 ± 7.08 ^a^	55.66 ± 5.57 ^c^	46.02 ± 4.60 ^d^	57.92 ± 5.79 ^c,d^	47.62 ± 4.76 ^d^	66.05 ± 6.60 ^a,b^	51.13 ± 5.1 ^c,d^	54.65 ± 5.46 ^c^	71.63 ± 7.16 ^a^

Different letters in the same row for each assay show significant differences among clones according to ANOVA with the Tukey test (*p* ≤ 0.05).

**Table 2:** Phenolic components of the seed flour from 10 clones of *Prosopis alba*.

Phytochemical Content	Total Phenolic (mg GAE/g Flour)	Non-Flavonoids Phenolics (mg GAE/g Flour)	Flavonoids (mg QE/g Flour)	Condensed Tannins (mg Pβ2E/100 g Flour)
P1	6.26 ± 0.44 ^b,c,d^	3.69 ± 0.38 ^a,b^	0.191 ± 0.002 ^b^	109.94 ± 1.37 ^e^
P3	5.05 ± 0.86 ^d^	2.68 ± 0.20 ^c^	0.111 ± 0.002 ^f,e^	78.12 ± 1.04 ^f^
P4	5.77 ± 0.47 ^b,c,d^	2.85 ± 0.29 ^b,c^	0.151 ± 0.008 ^c^	214.97 ± 2.37 ^b^
P5	5.22 ± 0.23 ^c,d^	3.14 ± 0.21 ^a,b,c^	0.137 ± 0.007 ^c,d^	194.15 ± 2.25 ^c^
P6	7.32 ± 0.36 ^a,b,c^	3.21 ± 0.27 ^a,b,c^	0.111 ± 0.014 ^f,e^	211.55 ± 0.94 ^b^
P7	8.58 ± 1.10 ^a^	3.95 ± 0.35 ^a^	0.116 ± 0.003 ^d,e,f^	168.54 ± 1.58 ^d^
P9	5.71 ± 0.50 ^b,c,d^	2.90 ± 0.26 ^b,c^	0.098 ± 0.003 ^f^	44.75 ± 0.79 ^g^
P10	7.65 ± 0.52 ^a,b^	4.01 ± 0.38 ^a^	0.308 ± 0.015 ^a^	219.53 ± 0.66 ^a^
P12	6.48 ± 1.37 ^a,b,c,d^	3.34 ± 0.32 ^a,b,c^	0.118 ± 0.010 ^d,e,f^	223.73 ± 1.86 ^a^
P13	5.99 ± 0.86 ^b,c,d^	3.25 ± 0.28 ^a,b,c^	0.131 ± 0.004 ^c,d,e^	222.49 ± 0.94 ^a^

Different letters in the same column for each assay show significant differences among clones according to ANOVA with the Tukey test (*p* ≤ 0.05).

**Table 3:** Antioxidant activity of the seed flour from 10 clones of *Prosopis alba*.

	Antioxidant Activity		
*P. alba* Clones	ABTS^•+^	H_2_O_2_	HO^•^
	SC_50_ (µg GAE/mL)	SC_50_ (µg GAE/mL)	SC_50_ (µg GAE/mL)
P1	4.0 ± 0.2 ^d^	5.9 ± 0.4 ^a^	3.8 ± 0.4 ^a^
P3	6.7 ± 0.4 ^a^	10.5 ± 0.3 ^b^	3.8 ± 0.4 ^a^
P4	5.0 ± 0.2 ^c^	13.6 ± 0.3 ^a^	4.4 ± 0.4 ^a^
P5	5.4 ± 0.2 ^b,c^	12.1 ± 0.3 ^a^	4.6 ± 0.5 ^a^
P6	6.0 ± 0.3 ^a,b^	20.7 ± 0.4 ^a^	4.2 ± 0.4 ^a^
P7	5.6 ± 0.3 ^b,c^	20.8 ± 0.3 ^b^	4.2 ± 0.4 ^a^
P9	5.7 ± 0.3 ^b,c^	23.3 ± 0.3 ^b^	4.0 ± 0.4 ^a^
P10	5.0 ± 0.2 ^c^	11.6 ± 0.3 ^b^	3.8 ± 0.4 ^a^
P12	5.0 ± 0.2 ^c^	17.8 ± 0.3 ^b^	3.7 ± 0.4 ^a^
P13	5.5 ± 0.3 ^b,c^	20.5 ± 0.4 ^a^	3.7 ± 0.4 ^a^

Different letters in the same column for each assay show significant differences among clones according to ANOVA with the Tukey test (*p* ≤ 0.05).

## Data Availability

Data is contained within the article or Supplementary Materials.

## Supplementary Materials

The following supporting information can be downloaded at: https://www.mdpi.com/article/10.3390/foods11182857/s1, Figure S1: HPLC chromatogram of polyphenols from *P. alba* seed flour (Clones P7–P13). Detection: UV_254_ nm. Compounds: 1: isoschaftoside hexoside; 2: schaftoside hexoside; 3: Vicenin II (Apigenin-di-*C*-hexoside)/Isomer; 4: Vicenin II/Isomer; 5: Isoschaftoside (Apigenin-*C*-hexoside-*C*-pentoside); 6: Schaftoside; 7: unknown 8: Vitexin; 9: Isovitexin; 10: unknown.

## Abbreviations

PEE: phenolic enriched extracts, GAE: gallic acid equivalent, QE: quercetin equivalent, PB_2_E: procyanidin B2 equivalent, HPLC-DAD: HPLC coupled to a diode array detector, SC_50_: 50% scavenging capacity, HRP: horseradish peroxidase, PCA: principal component analysis, HCA: hierarchical clusters (HCA).
