# Supplementary material for: Prosopis alba Seed as a Functional Food Waste for Food Formulation Enrichment
Source: Foods. 2022 Sep 15;11(18):2857. doi: 10.3390/foods11182857 (PMC9497860; doi:10.3390/foods11182857)
Supplement: Supplementary file 1 [file foods-11-02857-s001.zip › foods-1869817-supplementary.pdf]

Supplementary material

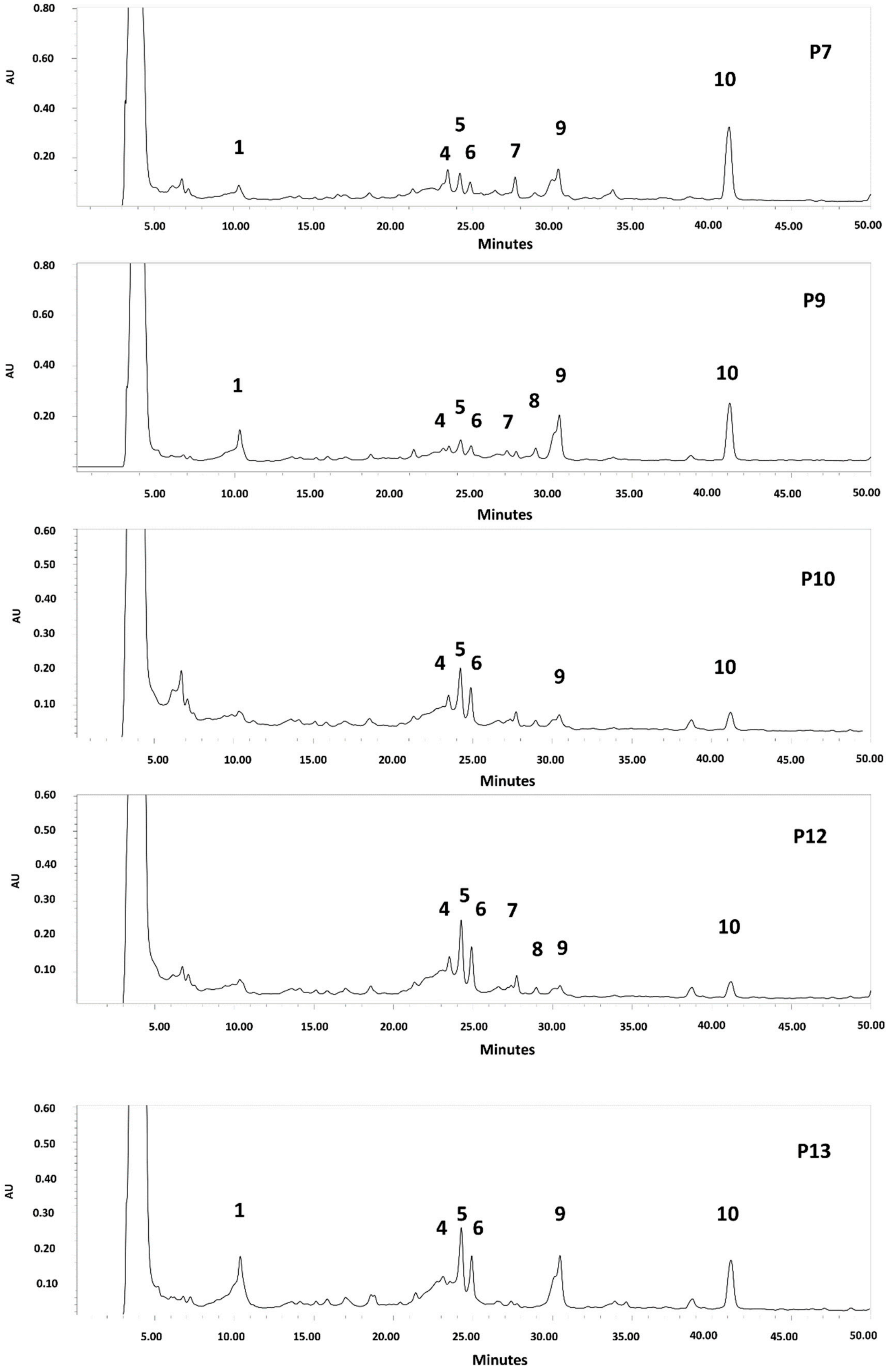

Figure S1. HPLC chromatogram of polyphenols from *P. alba* seed flour (Clones P7–P13). Detection: UV_{254} nm. Compounds: 1: isoschaftoside hexoside; 2: schaftoside hexoside; 3: Vicenin II (Apigenin-di-*C*-hexoside)/Isomer; 4: Vicenin II/Isomer; 5: Isoschaftoside (Apigenin-*C*-hexoside-*C*-pentoside); 6: Schaftoside; 7: unknown 8: Vitexin; 9: Isovitexin; 10: unknown.
